# Characteristics and Therapeutic Potential of Dental Pulp Stem Cells on Neurodegenerative Diseases

**DOI:** 10.3389/fnins.2020.00407

**Published:** 2020-05-07

**Authors:** Tomoyuki Ueda, Masatoshi Inden, Taisei Ito, Hisaka Kurita, Isao Hozumi

**Affiliations:** Laboratory of Medical Therapeutics and Molecular Therapeutics, Gifu Pharmaceutical University, Gifu, Japan

**Keywords:** dental pulp stem cells, stem cells from human exfoliated deciduous teeth, conditioned medium, neurodegenerative disease, cell therapy

## Abstract

To evaluate the therapeutic potential of stem cells for neurodegenerative diseases, emphasis should be placed on clarifying the characteristics of the various types of stem cells. Among stem cells, dental pulp stem cells (DPSCs) are a cell population that is rich in cell proliferation and multipotency. It has been reported that transplantation of DPSCs has protective effects against models of neurodegenerative diseases. The protective effects are not only through differentiation into the target cell type for the disease but are also related to trophic factors released from DPSCs. Recently, it has been reported that serum-free culture supernatant of dental pulp stem cell-conditioned medium (DPCM) contains various trophic factors and cytokines and that DPCM is effective for models of neurodegenerative diseases such as Alzheimer’s disease (AD), Parkinson’s disease (PD), and Amyotrophic Lateral Sclerosis (ALS). Moreover, the use of stem cells from human exfoliated deciduous teeth (SHEDs) has been considered. SHEDs are derived from deciduous teeth that have been disposed of as medical waste. SHEDs have higher differentiation capacity and proliferation ability than DPSCs. In addition, the serum-free culture supernatant of SHEDs (SHED-CM) contains more trophic factors, cytokines, and biometals than DPCM and also promotes neuroprotection. The neuroprotective effect of DPSCs, including those from deciduous teeth, will be used as the seeds of therapeutic drugs for neurodegenerative diseases. SHEDs will be used for further cell therapy of neurodegenerative diseases in the future. In this paper, we focused on the characteristics of DPSCs and their potential for neurodegenerative diseases.

## Dental Pulp Cells

Generally, the structure of the tooth consists of three layers (Chai et al., 2000; Shi and Gronthos, 2003). The dental pulp is located in the center of the tooth and is a fibrous connective tissue that fills the pulp cavity inside the tooth. It is located in the center of the tooth and is surrounded by dentin. Additionally, it supports the vitality of the tooth by supplying a variety of features via the apical foramen (e.g., blood vessels) and plays a key role in maintaining the tooth. Previous studies indicate that dental pulp contains a cell population with high self-renewal and cell proliferation abilities *in vitro* and can establish induced pluripotent stem (iPS) cells more efficiently than skin fibroblasts (Gronthos et al., 2000; Tamaoki et al., 2010, 2014). The regenerative abilities of dental pulp cells (DPCs) may be derived from these stem cells, known as dental pulp stem cells (DPSCs). To harvest stem cells from bone marrow is very difficult, because the invasive nature of the collection process can lead to physical complications for the donor as well as the recipient. On the other hand, dental pulp can be collected from sources such as discarded wisdom teeth, therefore reducing invasive effects on the body and decreasing the risk of harm to the donor (Geng et al., 2017; Xiao et al., 2017). Thus, DPCs have the potential to compensate for bone marrow collection difficulties. From an ethical viewpoint, DPCs are the ideal source of stem cells.

## Dental Pulp Stem Cells (DPSCs)

DPSCs were first isolated from DPCs in 2000 (Gronthos et al., 2000). They are characterized by their high clonal capacity, fibroblast-like morphology, and high proliferation rate. Additionally, nestin, vimentin, OCT-4, and SOX-2, which are all specific markers of undifferentiated embryonic stem cells, are expressed (Kiraly et al., 2009; Govindasamy et al., 2011; Sakai et al., 2012). Recently, DPSCs have attracted attention in the field of regenerative medicine, especially with regard to neurodegenerative diseases. It was shown that DPSCs can be differentiated into functionally active neuronal cells under neuronal differentiation conditions (Arthur et al., 2008; Kiraly et al., 2009). According to previous reports, these differentiated neurons have voltage-dependent sodium channels that play an important role in the generation of action potentials (Arthur et al., 2008). Other research groups have discovered that it is possible to differentiate DPSCs into other specific types, such as dopaminergic neurons (Kanafi et al., 2014; Singh et al., 2017; Gonmanee et al., 2018). Moreover, these stem cells have been shown to be involved in processes involving a variety of cell types including bone formation, cartilage formation, myogenesis, adipogenesis, and differentiation into neural lineages (Gronthos et al., 2002; Laino et al., 2005; d’Aquino et al., 2007; Stevens et al., 2008; Pisciotta et al., 2018). Thus, the application of DPSCs in regenerative medicine has been widely expected.

On the other hand, mesenchymal stem cells (MSCs), another stem cell type derived from tissue such as bone marrow, secrete various growth and neurotrophic factors. It is suggested that these cells are activated and tissue is regenerated by several neurotrophic factors. However, DPSC transplantation has been shown to lessen tissue injury in the brain (Nito et al., 2018). Studies also indicate that neurotrophic factors, such as glial cell line-derived neurotrophic factor (GDNF), neurotrophin-3 (NT-3), brain-derived neurotrophic factor (BDNF), and vascular endothelial growth factor (VEGF), promote neuroprotection and have a protective effect on the effectiveness of cell therapy against neurodegenerative diseases (Nosrat et al., 2004; Sakai et al., 2012). In addition, the expression of neurotrophic factors in DPSCs has been shown to be higher than that of MSCs derived from adipose tissue (Mead et al., 2014). Furthermore, other reports showed that DPSCs have protective effects in spinal cord injury models, Alzheimer’s disease (AD) models, and retinal injury models by releasing neurotrophic factors in both *in vitro* and *in vivo* experiments (Mead et al., 2013; Ahmed et al., 2016; Zhang et al., 2016). Judging from the reports, DPSCs differentiate into target cells, and the neurotrophic factors released from DPSCs can be used in cell therapy.

Culture medium collected from cells in a culture, also known as a conditioned medium (CM), contains small molecule metabolites and growth factors released from cells. CM cultured for an appropriate period of time includes a variety of cell-derived factors. For example, stromal stem cells and adipose-derived MSCs are known to release various growth factors such as epidermal growth factor (EGF), cytokines, and insulin-like growth factor-1 (IGF-1) (Osugi et al., 2012; Gehmert et al., 2014). In addition, several factors secreted by DPSCs play a key role in neuronal survival, proliferation, and differentiation Nosrat et al. (2001, 2004) and Arthur et al. (2009). Recently, it has been revealed that dental pulp conditioned medium (DPCM), which is a serum-free culture supernatant of DPCs, has many therapeutic effects and factors such as cytoprotective effect, revascularization factor, and fibrosis inhibitory factor (Matsubara et al., 2015; Mita et al., 2015). In our previous research, DPCM contained many neurotrophic factors such as NT-3 and nerve growth factor (NGF) and contributed to neuroprotective effects against neurodegenerative diseases. In particular, DPCM reduced neuronal cell death due to endoplasmic reticulum stresses and oxidative stresses (Kudo et al., 2015). In addition, some reports showed that the pathological condition is remarkably improved by intravenous administration of DPCM to refractory acute and chronic inflammatory disease model animals such as those of cerebral infarction, spinal cord injury, diabetes, renal disorder, and interstitial pneumonia (Inoue et al., 2013; Hattori et al., 2015; Izumoto-Akita et al., 2015). Therefore, it is obvious that even if DPSCs are not transplanted, treatments with CM containing the factors released from DPSCs alone exert therapeutic effects against neurodegenerative diseases.

## Stem Cells From Human Exfoliated Deciduous Teeth (SHEDs)

There is another type of dental pulp stem cell called stem cells from human exfoliated deciduous teeth (SHEDs), which is specifically found in the dental pulp of deciduous teeth. In 2003, stem cells from human exfoliated deciduous teeth (SHEDs) were isolated from deciduous teeth for the first time. They express early mesenchymal and neuroectodermal stem cell markers (Miura et al., 2003). Like bone marrow MSCs (BMSCs), SHEDs can differentiate into various cells such as chondrocytes, adipocytes, endothelial cells, and functionally and structurally active neurons *in vitro* (Miura et al., 2003). The growth rate of SHEDs has been shown to be much higher than the growth rate of DPSCs, and a comparison of gene expression analysis results indicated that 4386 genes were expressed; two times the amount in DPSCs (Nakamura et al., 2009). Furthermore, several reports showed that transplantation of SHEDs as well as of DPSCs promoted recovery from central neuropathy in a paracrine manner via the release of various trophic factors (Inoue et al., 2013; Yamagata et al., 2013). Such transplantation may be effective for neurodegenerative diseases since SHEDs are differentiated into functionally active neuronal cells such as dopaminergic neurons (Fujii et al., 2015). In addition, the serum-free culture supernatant of SHEDs (SHED-CM) as well as that of DPSCs is expected to have protective effects. SHED-CM includes a variety of growth factors such as hepatocyte growth factor (HGF) at levels much higher than in BMSC-CM (Matsubara et al., 2015). HGF in SHED-CM has a protective effect against apoptosis induced by ischemia (Yamagata et al., 2013). The protective effect of SHED-CM has also been confirmed in AD model mice (Mita et al., 2015). It is not always easy to recover naturally exfoliated deciduous teeth immediately in order to start a SHED culture, compared to permanent (wisdom) teeth extracted in clinics.

## Neurodegenerative Disease

### Alzheimer’s Disease

Alzheimer’s disease (AD) is a progressive dementia that accounts for the highest frequency of dementia cases in elderly persons. Early symptoms often include short-term memory impairment, language dysfunction, poor judgment, and behavioral disorder. The most obvious pathologies are extracellular β-amyloid deposition and intracellular neurofibrillary tangles. Both pathologies lead to synapse and neuronal loss, resulting in brain atrophy. In spite of the effects of many candidate compounds having been confirmed in clinical trials, only three cholinesterase inhibitors and memantine have been used at an international level (Szeto and Lewis, 2016). However, their clinical effect is inadequate. They are used as symptomatic therapy and do not treat the underlying cause of AD. Recently, disease-modifying therapies have been focused on as one of the new treatments for AD (Cummings et al., 2016). Cell therapy using DPSCs and SHEDs may have the potential to improve AD pathology. In *in vitro* AD models, DPSCs release NGF, GDNF, BDNF, and other neurotrophic factors, reducing amyloid β-induced toxicity (Apel et al., 2009) and protecting against okadaic acid-induced model cells of AD (Wang et al., 2017). Moreover, in *in vivo* AD models, the transplantation of SHEDs improves the symptoms of AD mice due to their paracrine effect (Mita et al., 2015).

### Parkinson’s Disease

Parkinson’s disease (PD) is a neurodegenerative disease that causes the progressive loss of midbrain dopamine neurons. Resting tremor, rigidity, and bradykinesia are common symptoms. As symptoms progress, it becomes difficult for patients to work, and they may use a wheelchair or become bedridden. Most people diagnosed with PD are middle-aged, with the majority being 65 years of age or older. The cause is still unknown, but one hypothesis is that the protein α-synuclein aggregates and accumulates in dopamine neurons, resulting in the weakening of surrounding cells. Currently, in clinical practice, the use of L-DOPA or levodopa (dopamine replacement therapy) is most often used in the treatment of PD. However, this is only a symptomatic treatment and does not suppress the loss of dopamine neurons. In addition, most PD patients who receive intermittent L-dopamine therapy develop drug-induced dyskinesia (Lange and Lozano, 1998; Olanow and Koller, 1998). Utilization of DPSCs may be expected to be beneficial for PD. *In vitro*, neurotrophic factors such as NGF and GDNF released by DPSCs protect midbrain neurons damaged by 6-hydroxydopamine (6-OHDA), a selective dopaminergic toxin (Apel et al., 2009), and exosomes released from DPSCs protect midbrain neurons from 6-OHDA-induced apoptosis (Jarmalavičiūtë et al., 2015). In addition, it has been shown that DPSCs play a protective role by inhibiting NO production in a co-culture of dopamine neurons and microglia (Gnanasegaran et al., 2017). *In vivo*, the paracrine effect of neurotrophic factors from SHEDs demonstrates neuroprotection against neurodegeneration and recovery of the nigrostriatal dopamine neurons in model rats from 6-OHDA-induced degeneration (Fujii et al., 2015; Zhang et al., 2018). However, the effect of DPSCs on α-synuclein aggregates under the pathogenesis of PD is still unknown. Therefore, it is necessary to continue basic research on these aggregates on DPSCs for future clinical application.

### Amyotrophic Lateral Sclerosis

Amyotrophic Lateral Sclerosis (ALS) is a progressive neurodegenerative disease. Usually, it begins with a decrease in limb motor function, and soon, it becomes difficult to talk, eat, or drink. Eventually, the patient dies due to respiratory muscle paralysis. Symptomatic treatments such as riluzole and edaravone are most commonly used, but the clinical benefits are limited. Stem cells, including DPSCs and SHEDs, have received great attention for ALS, for which no effective therapeutics have been developed (Mazzini et al., 2019). Several reports have demonstrated that DPCM suppresses neuromuscular junction fragility and motor neuron loss in ALS mouse models (Galán et al., 2010; Wang et al., 2019). DPSCs and SHED will be promising candidates for the treatment of ALS, because they may also have miraculous effects that we do not yet know of.

## Other Diseases

Spinal cord injury is usually caused by an accident or a fall, resulting in a disorder in motor or sensory function. Once injured, the spinal cord does not recover function. When the spinal cord is injured, late effects such as paralysis of the limbs may remain, which may cause serious problems in daily life. The progression of a spinal cord injury is closely related to the spread of inflammation at the site of injury. There are two types of macrophages, M1 and M2. M1 macrophages digest debris and are involved in inflammation. M2 macrophages, on the other hand, function to reduce inflammation. M1 macrophages induce inflammation by releasing pro-inflammatory cytokines and nitric oxide. M1 macrophages also play a key role in astrocyte recruitment and activation. On the other hand, M2 macrophages promote tissue repair by secreting anti-inflammatory cytokines. As a result, M2 macrophages promote axonal outgrowth and oligodendrocyte progenitor cell proliferation. In previous studies, transplantation of DPSCs in model mice after spinal cord injury released various trophic factors, such as BDNF and GDNF, and markedly improved motor function (Zhang et al., 2016). In addition, transplantation of SHEDs into the spinal cord injury model showed significant locomotor function recovery (Taghipour et al., 2012). Moreover, the secreted ectodomain of sialic acid-binding Ig-like lectin-9 (ED-Siglec-9) and monocyte chemoattractant protein-1 (MCP-1), which are specifically contained in SHED-CM, promote marked functional recovery in a rodent spinal cord injury model by inducing an M2-dominant neurorepairing microenvironment (Matsubara et al., 2015).

Multiple sclerosis (MS) a demyelinating disease of the central nervous system. MS is prevalent in Europe, and in some parts of Northern Europe, there are more than 100 patients per 100,000 population. MS mostly affects young adults, with an average age of onset of around 30 years. The cause of MS is not yet clear, but it is thought to result from immune abnormalities. The main symptoms include impaired vision, nystagmus, difficulty swallowing, and difficulty talking. If the cerebellum is impaired, you will not be able to walk straight, and your hands will shake. Cerebral lesions can affect cognitive function as well as causing sensory and motor disorders of the limbs. The protective effect of SHED or SHED-CM has been shown in the treatment of experimental autoimmune encephalomyelitis (EAE), which Is one of the models of MS. Administration of SHED or SHED-CM reduced the expression of inflammatory cytokines in the spinal cord, suppressed demyelination, and improved EAE clinical scores (Shimojima et al., 2016; Rossato et al., 2017). These results suggest that the anti-inflammatory properties of DPSC and SHED could be candidates for the treatment of both spinal cord injury, MS, and neurodegenerative diseases.

## Perspective

Currently, autologous stem cell transplantation is the most extensively used cell therapy treatment in mainstream medicine. However, the collection and use of a patient’s own autologous stem cells is associated with several risks, such as huge costs in money and time. Thus, there are several advantages to using another person’s cells that have been evaluated for safety and function beforehand. Recently, allogeneic stem cell transplantation research has begun in autologous cells for several diseases. Human leukocyte antigens (HLAs) play an important role in allogeneic transplantation. HLAs are a type of protein that is distributed in almost all cells and distinguishes cells from each other. When a cell or organ is transplanted from a “non-self” HLA-type, an HLA type different from its own, the body recognizes it as a “foreign substance,” and a rejection reaction occurs. Statistically, when the HLA types of 10,000 cells were analyzed, eight haplotype-homozygous cells were revealed. This appears to cover half of the Japanese population. In addition, when 200,000 cells were analyzed, 65 haplotypes homozygous were revealed, covering 90% of the Japanese population. Considering the present situation, further utilization of DPSCs such as dental pulp banks is expected and will be crucial for the future of regenerative medicine.

**FIGURE 1:**
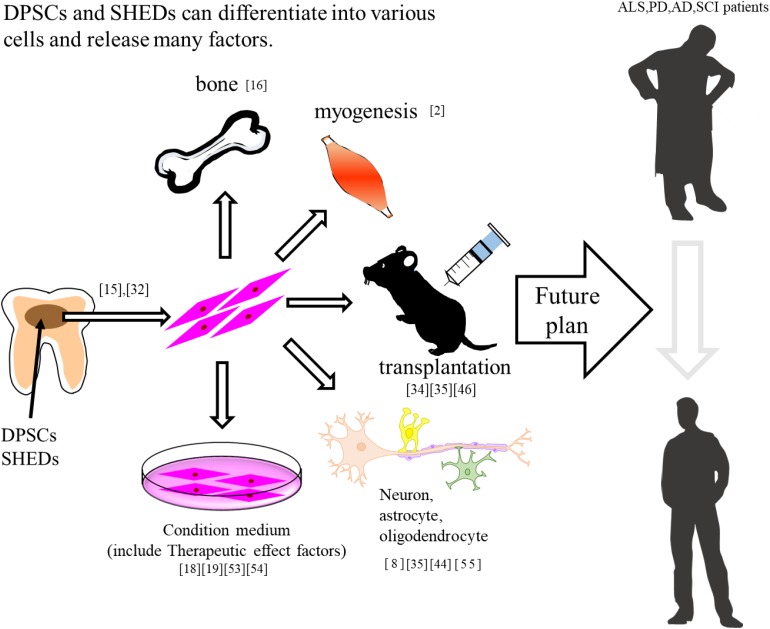
DPSCs and SHEDs differentiate into bone, muscle, and neuron. In addition, these have therapeutic effects on neurodegenerative diseases.

DPSCs are considered clinical waste and are very safe for transplantation. In addition, from the perspective of having a patient use their own wisdom and deciduous teeth, the utilization of DPSCs has no ethical issues. It is known that dental pulp stem cells have a powerful regenerative potential that is different from other stem cells (e.g., umbilical cord blood and bone marrow) and have been shown to be useful biological stem cells for the treatment of various diseases. In the field of the central nervous system, it has been reported that transplanted DPSCs act in a paracrine manner by releasing neurotrophic factors. Recently, SHED-CM has been reported to be more effective than DPCM. SHED-CM is supposed to contain more trophic factors, cytokines, and biometals. SHED-CM is also considered to be one of the useful treatments for some neurodegenerative diseases such as AD, PD, and ALS (Table 1). In the future, if the therapeutic effects and transplantation safety can be confirmed by using a primate model more closely related to humans, it will be an effective candidate treatment for diseases for which there are currently no effective treatments (Figure 1).

**TABLE 1:** Therapeutic potential of dental pulp stem cells and stem cells from human exfoliated deciduous teeth on various diseases such as neurodegenerative disease.

**Type**	**For disease**	**Differentiated status**	**Role**	**References**
DPSCs	Parkinson’s disease (PD)	Dopaminergic cell-type differentiated	DPSCs differentiate efficiently into functional dopaminergic cell type	Kanafi et al., 2014; Gonmanee et al., 2018
		Dopaminergic neurons differentiated	DPSCs have immunomodulatory capacities and were indicated applicability for cell replacement therapy (CRT)	Singh et al., 2017
		Dopaminergic neurons differentiated	Dental pulp cells provide neurotrophic factor and differentiate into neurons	Nosrat et al., 2004
		Undifferentiated	DPSCs release neurotrophic factors such as NGF and GDNF and protect midbrain neurons against 6-hydroxydopamine (6-OHDA) induced celltoxicity	Apel et al., 2009
		Undifferentiated	exosomes released from DPSCs provide midbrain neurons damaged by 6-OHDA from apoptosis	Jarmalavičiūtë et al., 2015
		Undifferentiated	DPSCs play a protective role through inhibiting NO production in a co-culture of dopamine neurons and microglia	Gnanasegaran et al., 2017
	Alzheimer’s disease (AD)	Undifferentiated	DPSCs secreted many neuroprotective factor such as vascular endothelial growth factor (VEGF). fins-related tyrosine kinase 3 (FLT-3). and monocyte chemotactic protein 1 (MCP-1). etc	Ahmed et al., 2016
		Undifferentiated	DPSCs secreted NGF, GDNF. BDNF and other neurotrophic factors, reducing amyloid β-induced toxicity	Apel et al., 2009
		Undifferentiated	DPSCs protect against okadaic acid-induced model cells of AD	Wang et al., 2017
	Amyotrophic lateral sclerosis (ALS)	Conditioned medium	DPCM suppress neuiomuscular junction fragility and motor neuron loss in models of ALS	Wang et al., 2019
	Spinal cord injury (SCI)	Undifferentiated	DPSCs have protective effects by releasing neurotrophic factors	Mead et al., 2013
		Undifferentiated	DPSCs secreted many neuroprotective factor such as BDNF, GDNF. b-NGF, and NT-3	Zhang et al., 2016
	Middle cerebral artery occlusion	Undifferentiated	Transplantation of DPSCs inhibited microglial activation and pro-inflammatory cytokine expression	Nito et al., 2018
	diabetes	Conditioned medium	SHED-CM provides direct protection and encourages the propagation of β-cells	Izumoto-Akita et al., 2015
	Differentiation into others cells	Bone formation, cartilage formation. Myogenesis, adipoenesis neural lineage	DPSCs differentiate efficiently into some kinds of cells	Gronthos et al., 2002; Laino et al., 2005; d’Aquino et al., 2007; Stevens et al., 2008; Pisciotta et al., 2018
SHEDs	PD	Undifferentiated	SHEDs is differentiated into a functionally active neuronal cells such as dopamine neurons	Fujii et al., 2015; Zhang et al., 2018
	AD	Conditioned medium	SHED-CM attenuated the pro-inflammatory responses induced by β-amylold plaques	Mita et al., 2015
	SCI	Undifferentiated	Transplantation of SHEDs into the model of spinal card injury showed locomotor functional recovery	Taghipour et al., 2012
	SCI	Conditioned medium	SHED-CM improved the model of spinal cord injury though inducing an M2-dominant neurorepairing micro environment	Matsubara et al., 2015
	Middle cerebral artery occlusion	Conditioned medium	SHED-CM promoted the migration and differentiation of endogenous neuronal progenitor cells.	Inoue et al., 2013;
	Perinatal hypoxia-ischemia	Conditioned medium	Conditioned medium inhibited apoptosis. and reduced tissue loss.	Yamagata et al., 2013
	Others	Undifferentiated	SHEDs differentiate into osteoblasts, chondrocytes, endothelial cells.	Miura et al., 2003

## Author Contributions

TU, MI, TI, HK, and IH wrote the manuscript.

## Conflict of Interest

The authors declare that the research was conducted in the absence of any commercial or financial relationships that could be construed as a potential conflict of interest.
